# Levothyroxine sodium loaded dissolving microneedle arrays for transdermal delivery

**DOI:** 10.5599/admet.1317

**Published:** 2022-09-13

**Authors:** Riyam F. Ghazi, Mohammed H. Al-Mayahy

**Affiliations:** Department of Pharmaceutics, College of Pharmacy, Mustansiriyah University, Baghdad-Iraq

**Keywords:** Hyaluronic acid, levothyroxine sodium, dissolving microneedles, micromoulding, transdermal delivery

## Abstract

Levothyroxine (LT-4) sodium has shown variable bioavailability following oral administration. This can be assigned to the significant influence of gastrointestinal conditions, food and drugs administered concomitantly on the rate and extent of absorption from the gastrointestinal tract. Thus, the aim of this research study was to establish an efficient transdermal delivery system of LT-4 sodium via the application of hyaluronic acid dissolving microneedles. Microneedles-based drug delivery system consists of sharp-tip needles that puncture the top layers of the skin in a minimally invasive manner to create physical channels through which therapeutic molecules can easily diffuse into/across the skin. Hyaluronic acid polymer at different ratios (5-60 %) was used to prepare microneedle arrays (100 needles per array) using a micromoulding technique. Characterisation tests were carried out to select the optimum formulation. F11 formula containing 50% w/v hyaluronic acid and 1% v/v Tween 80 formula showed an appropriate needle shape with dimensions of 432 ± 6.4 μm in height and a tip diameter of 9.8 ± 1.3 μm. The microneedle arrays demonstrated a suitable mechanical strength after applying a force of 32 N per array and an excellent insertion ability both in Parafilm M® and human skin. The in vivo dissolution of microneedles was started rapidly within 5 minutes following the insertion in the skin and completed at 1 hour. Ex vivo permeation study using human skin has shown a significant improvement in LT-4 sodium delivery across the skin compared to control preparations (drug solution and microneedle free film). The microneedle array F11 has significantly (P ≤ 0.05) increased LT-4 sodium permeation through the skin (cumulative permeated amount of 32 ± 2 μg/cm^2^) in comparison to the control solution (cumulative permeated amount of 0.7 ± 0.07 μg/cm^2^) and the microneedle free film (cumulative permeated amount of 0.1 ± 0.02 μg/cm^2^) over 7 hours. The findings from the irritation test revealed that mild erythema was produced from the application of microneedle arrays which disappeared within 24 hours. Accordingly, dissolving hyaluronic acid microneedles could be a feasible and effective approach to delivering LT-4 sodium transdermally without causing significant skin damage.

## Introduction

Levothyroxine (LT-4) sodium is predominantly used as replacement therapy for the treatment of hypothyroidism, chronic lymphocytic thyroiditis and simple non-endemic goiters. It is administered in small doses (micrograms), which provides a greater opportunity for clinically significant interactions through the absorption phase to occur [1]. Several gastrointestinal conditions such as celiac disease, atrophic gastritis, lactose intolerance and pyloric infection in *Helicobacter* could limit the oral absorption of LT-4 sodium [1,2]. Simultaneous administration of oral drugs and food intake can significantly affect LT-4 bioavailability, including the onset, rate and extent of drug absorption. Thyroxine malabsorption has been noticed following jejunoileal bypass procedures, in short-bowel syndrome, in association with vitamin B12 malabsorption, in severe hepatic cirrhosis, as well as in congestive heart failure [3]. Therefore, it is required to develop an alternative delivery system for a more efficient administration of LT-4 sodium, for instance, via the transdermal route. However, LT-4 is regarded as a poor candidate for transdermal drug delivery due to its inappropriate permeability characteristics. Thus, an active skin permeation enhancement strategy is needed for its delivery across the skin, such as microneedle arrays.

Microneedles (MNs) represent a unique technology for enhancing the drug permeability through the *stratum corneum*. MNs based drug delivery consists of sharp-tip needles that puncture the top layers of the skin in a minimally invasive manner to create physical channels through which therapeutic molecules can easily diffuse into/across the skin [4,5]. Typically, this approach uses 1 to 400 needles (100 μm to 1 mm apart) which may range from 150 μm to 1000 μm in length with a diameter from 50 μm to 80 μm. The MN array is placed on the skin surface such that MNs penetrate the *stratum corneum* layer, bypassing it and delivering therapeutic agents into the viable epidermis directly. Since the epidermis has no nociceptors (pain receptors) and the MNs are frequently do not reach the dermis, pain sensation is not triggered and therefore, they are regarded as a minimally invasive technique with pain-free delivery. Morphologically microneedles can be classified into five different types, including solid microneedles [6], coated microneedles [7], dissolving microneedles [8], hollow microneedles [9] and hydrogel-forming microneedles [10]. They can be manufactured of a variety of materials, such as metals [11], inorganic materials [12] and polymeric materials [13].

Drug-loaded dissolving MNs containing a drug within the needle matrix can overcome the limitations associated with other MN types. They solve the problem of the two-step application of solid MNs. As a result, a one-step application technique will be available [14]. In comparison to coated MNs, a higher and more precise drug loading can be achieved by dissolving MNs [15]. Additionally, they eliminate the need for specialized techniques used in hollow MNs to control the flow rate and pressure for driving the flow of liquid into the skin. However, some of the biodegradable polymers, including PLGA, polylactic acid, polyglycolic acid and polycarbonate used in the manufacturing of dissolving MNs are unsuitable for the fabrication of LT-4 sodium loaded MNs, particularly during the manufacturing process. This can be attributed to the need for a heating step that may cause a degradation of heat-sensitive drugs such as LT-4 sodium. Other materials used in the fabrication of dissolving MNs, such as carbohydrates, undergo hydrolysis in a moist environment at a humidity level exceeding 43 %, resulting in needle distortion or disappearance, as well as insufficient insertion ability into the skin [16,17].

Hyaluronic acid (HA), commonly utilized in skincare products, was identified to generate MNs with good biocompatibility and deformation resistance. The obtained HA MNs were easily prepared without the need of a heating step. In addition, they have sufficient strength to reliably pierce the skin, dissolve and rapidly release the contained drug within the skin [18,19]. Therefore, in the present study, novel LT-4 sodium-loaded HA MNs were developed for enhancing transdermal delivery. To the best of our knowledge, this is the first study conducted to investigate LT-4 delivery across the skin using HA MNs.

## Experimental

### Materials

Levothyroxine sodium and hyaluronic acid 20 KD were purchased from (Look Chemical, China). Tween 80 was obtained from (Chemical point, United Kingdom). Blue Silica gel powder was purchased from (Om Chemicals, India) and Formalin was obtained from (Sigma-Aldrich, Germany). All chemicals and reagents used were of analytical grade.

### Preparation of LT-4 sodium loaded hyaluronic acid microneedle arrays

MN arrays were manufactured by micromoulding technique with HA as the structural material. MNs manufacturing can be considered as a transcription process from the micromould [20]. Initially, LT-4 sodium was dissolved in deionised water (DW) with the aid of Tween 80. Following this, HA was added to this solution at specific percentages and the volume was completed up to 100 % with DW to prepare different polymeric formulation mixtures as illustrated in Table 1. The mixtures were then stirred for 15 minutes using a magnetic stirrer at 600 rpm and left to settle overnight to remove any bubbles formed. Afterward, 100 μL of each polymeric formulation mixture containing 50 μg of LT-4 as an active pharmaceutical ingredient was poured into MN moulds (PDMS mould consisting of 10 × 10 array on a 0.64 cm^2^ area with 500 μm needle height, 300 μm needle base and 500 μm interspacing). The MN moulds were subjected to centrifugation for 40 minutes for two rounds (2 × 40 min.) to ensure the whole volume of polymeric mixture has entered microcavities of the mould. During the centrifugation process, the MN moulds were covered with silicon caps and adhesive tape to prevent the solution spill out from the mould. The moulded mixture was kept to dry for 24 hours in a desiccator at ambient conditions protected from light. The microneedle arrays were then removed from the mould and examined using a digital microscope. MNs were stored wrapped with aluminium foil in a desiccator until use.

### Characterisation of hyaluronic acid microneedle arrays

#### Assessment of the mechanical properties of HA microneedle arrays

A TA-XT2 Texture Analyser (Stable Microsystems, Copley, UK) was used to test MN arrays for their mechanical strength. In brief, MN arrays were mounted to the Texture Analyser's movable probe (0.5 cm in length). The probe was set to move downward at a pre-test speed of 1 mm/s, test speed of 0.5 mm/s and post-test speed of 10 mm/s. A force of 32 N per array was applied for 30 seconds to press the MN arrays down towards a flat metal block of 9.2 × 5.2 cm dimensions [18]. A digital microscope (RoHs, China) was utilised to observe the MN arrays prior and following the appliance of the compression load. Individual MNs’ heights were measured before and after testing using the ruler feature of Image J® software (US National Institutes of Health, Bethesda, Maryland, USA) in order to determine the percent height reduction using equation number (1) [21]:


(1)

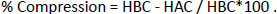



HBC is the height prior to compression and HAC is the height following compression.

#### *In vitro* drug release study

*In vitro* drug release of LT-4 sodium was performed for MNs formulations (F8, F9, F10 and F11) using Franz diffusion cells with an exposed surface area of 0.785 cm^2^. MN arrays were fixed on a small sieve and mounted on Franz cells with the needles facing downwards. The receptor chamber was filled with 10 mL of the solvent mixture containing ethanol (25 % v/v) and DW (75 % v/v) as a receptor fluid. Franz cells were then placed in diffusion cells apparatus (stirring water bath, Orchid Scientific, India) at 37 °C. The receptor fluid was stirred continuously by a small Teflon-coated magnetic bar at 400 RPM. Sink condition was maintained; since the samples of 1 mL were removed from the receptor, fluid was recovered with the same volume of the fresh pre-heated solvent mixture. Samples withdrawn were analyzed for LT-4 sodium content by UV-spectrophotometer to determine the percentage of drug released [22].

#### Detection of microneedles insertion ability using Parafilm M®

In their latest work (Larraneta *et al*., 2014), they demonstrated a great resemblance between Parafilm M® (PF) layers and porcine skin, suggesting that simple insertion tests on PF layers may be performed reliably. The film is folded into eight layers to achieve a thickness of approximately 1 mm, with one layer being 127 μm. The MN array was inserted into the eight membrane layers using a force of (32 N) generated by the texture analyser instrument. Following insertion, the MN array was withdrawn from the workbench and the PF layers were unfolded, allowing the number of holes remaining in each layer to be determined (using a digital microscope). The thickness of one layer being 127 μm ± 7 μm, hence the insertion depth can be deduced [23].

#### Insertion of microneedle arrays into excised human skin

To investigate the skin insertion ability of MN arrays prepared with different proportions of HA to penetrate the skin and form holes, an insertion and staining test was performed. Human skin samples were obtained from 32 years old female after liposuction surgery. MN arrays were inserted into human skin by pressing them against the skin surface for 1 minute and then removed. Subsequently, the skin surface was subjected to staining by application of methylene blue dye for 10 minutes to identify the insertion sites. After that, the excess of the dye was removed by tissue paper and the MN treated skin area was observed using a digital microscope. The insertion ratio for each MN array was calculated as the number of blue spots on the skin surface divided by the number of needles in the array [16].

#### Histological examination of microneedles treated skin

To demonstrate the penetration depth of MNs, a histological examination was performed. Skin cross-sectioning and staining techniques were used to assess the formation of microchannels within skin layers. Briefly, human skin samples treated with MNs were embedded in paraffin wax blocks and subjected to cross-sectioning into 5 μm slices using an electrical microtome. These cross-sectioned skin samples were then stained with hematoxylin and eosin (H&E) dyes to be examined under a light microscope [24].

#### *In vivo* dissolution study

*In vivo* dissolution study of the F11 MN array was performed using 2 month old rats weighing approximately 147-150 g (n = 3). The MNs arrays were inserted in the rat skin for different time intervals of 0, 5, 10, 20, 30 and 60 minutes. After that, the MN arrays were removed at each specific time point to be examined under a light microscope [25]. Ethical approval for all animal studies in this research was obtained from the Animal Ethics Committee (AEC) of the College of Pharmacy/ Mustansiriyah University/ Iraq-Baghdad under license no. 9.

#### Visualization of microneedles by scanning electron microscopy (SEM)

The morphological characteristics and dimensions of the MNs were determined using a Tescan Mira 3 SEM instrument. In this test, a MN array was fixed on an aluminum plate with adhesive tape and covered with gold/palladium of 20 μm thickness. The accelerating voltage was 15 kV and the time of the test was 2 minutes. A MN array was tested by SEM at different magnifications; 50, 100 and 500 X, to show the needles’ length, width, needle tip diameter and distance between needles [17]. The SEM images were captured using Tescan’s Essence software.

#### *Ex vivo* permeation study

Franz diffusion cells with an exposed surface area of 0.785 cm^2^ were used to investigate the permeation of LT-4 sodium from the optimum MN array formula F11 across human skin. Skin samples were obtained from the abdomen of 32 years old woman who had undergone a plastic surgery operation [19]. A consent form was obtained from the patient to collect and use her excised skin in *ex vivo* experiments. The skin was washed with normal saline and dried using tissue paper. After that, skin samples were wrapped in an aluminum foil and stored at -20 °C until required. Skin samples were used within a month of storage. Prior to starting the experiment, skin samples were soaked for 1 hour in normal saline to hydrate them and then they were placed on a flat desk with the *stratum corneum* facing upwards. Subsequently, HA MN arrays were physically pressed onto the skin for 30 seconds and secured using adhesive tape clamped between the donor and receptor chambers. The receptor chamber was filled with 10 mL of solvent mixture (ethanol 25 % v/v and DW 75 % v/v) as a receptor fluid. Franz cells (n = 6) were then placed in diffusion cells apparatus at 37 °C. The receptor fluid was stirred continuously by a small Teflon-coated magnetic bar at 400 rpm. Samples of 1 mL were withdrawn from the receptor fluid and replaced with a pre-heated fresh medium following each sampling time (10, 30, 60, 90, 180, 300 and 420 minutes) to maintain the sink condition.

The withdrawn samples were then assayed by a UV spectrophotometer to determine the concentration of LT-4 sodium. For comparison, control formulas containing the same ingredients of the optimum formula, one in a liquid form and another one as a film without needles (needle-free), were prepared to highlight the influence of needles on LT-4 sodium permeation. The control formulas were tested for skin permeation under the same experimental conditions as the HA MN arrays. The cumulative amount of LT-4 sodium was plotted against time to calculate the flux and percentage of the dose delivered across the skin [26].

#### Skin irritation test

Draize test was chosen to notice erythema and edema on the rat skin following the application of MN arrays [27]. Three healthy rats were selected and their skin was determined to be normal in the absence of a scratch or wound. Twenty-four hours before the test, the rats’ back skin was shaved. After that, the MN arrays were fixed to the skin for two hours. Following the removal of the MN arrays, Draize dermal scoring criteria were used to evaluate the erythema and edema at 1, 24 and 72 hours [27]. The following equation was used to estimate the Primary Irritation Index (P.I.I.):


(2)

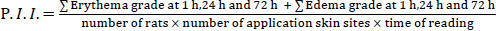



Draize dermal scoring criteria were used to evaluate the irritancy potential of the MN arrays, as described in Table 2. Additionally, a traditional hypodermic syringe of gauge 23 G was also used for the comparison with MNs for producing erythema and edema. These irritation signs were evaluated at 1, 24 and 72 hours using a digital microscope.

Statistical analysis was carried out using one-way ANOVA followed by the Tukey test to determine the presence of any significant difference among the data. All data are presented as the mean ± SD with *P* values of ≤ 0.05 being regarded as statistically significant [5].

## Results and Discussion

### Preparation, visual inspection and microscopic examination of levothyroxine sodium loaded hyaluronic acid microneedle arrays

LT-4 sodium-loaded HA MNs were prepared using micromoulding technique. HA was used as a matrix polymer in this study for reasons other than its biocompatibility and biodegradability; the manufacturing of HA MNs does not necessitate extreme conditions such as high temperatures or photolithography. Moreover, the application of HA dissolving MNs can improve the transport of active agents into and through the skin while also delivering HA into the skin itself as an added advantage [22]. As a result, the chosen micromoulding method with HA as a matrix polymer is ideally suited for incorporating heat and photosensitive molecules such as LT-4 sodium into MNs [28].

Not only does the MNs matrix material affect the effectiveness of the MNs, but it also does the geometry of MNs. In case the needle arrays were too close together, a "bed of nails" situation occurs, resulting in penetration failure [29]. Furthermore, numerous publications in the literature have linked the shape of needles ranging from conical, octagonal, rectangular, cylindrical and pyramidal to the required penetration force [30]. Typically, a pyramidal shape exerted the greatest puncture strength, making application easier [5]. The pyramidal design, according to Laue *et al*., enabled increased drug release by expanding the contact area with skin [31].

In this study, HA was used in different ratios ranging from 5 % to 60 % w/v to investigate the appropriate percentage for producing MNs with sufficient mechanical strength required for effective penetration into the skin. The drying time is also a crucial consideration in MNs manufacturing affecting the needle shape and the mechanical strength [32]. The drying time varies relatively depending on the concentration of HA. In general, 24 hours were required to obtain completely dry MN arrays in this study which is a suitable duration of time to be fitted with the large-scale production. Total dryness of the MNs was required to guarantee adequate mechanical strength [23]. On the other hand, inappropriate drying conditions led to wet MN arrays, which bent when removed from the mould resulting in unsatisfactory skin penetration capabilities. Tween 80 was added to enhance the solubility of LT4 sodium. In addition to enhancing solubility, Tween 80 plays a role in providing the formulation with the required elasticity necessary for the easy removal of MNs from the mould without any fracture.

**Figure 1.**
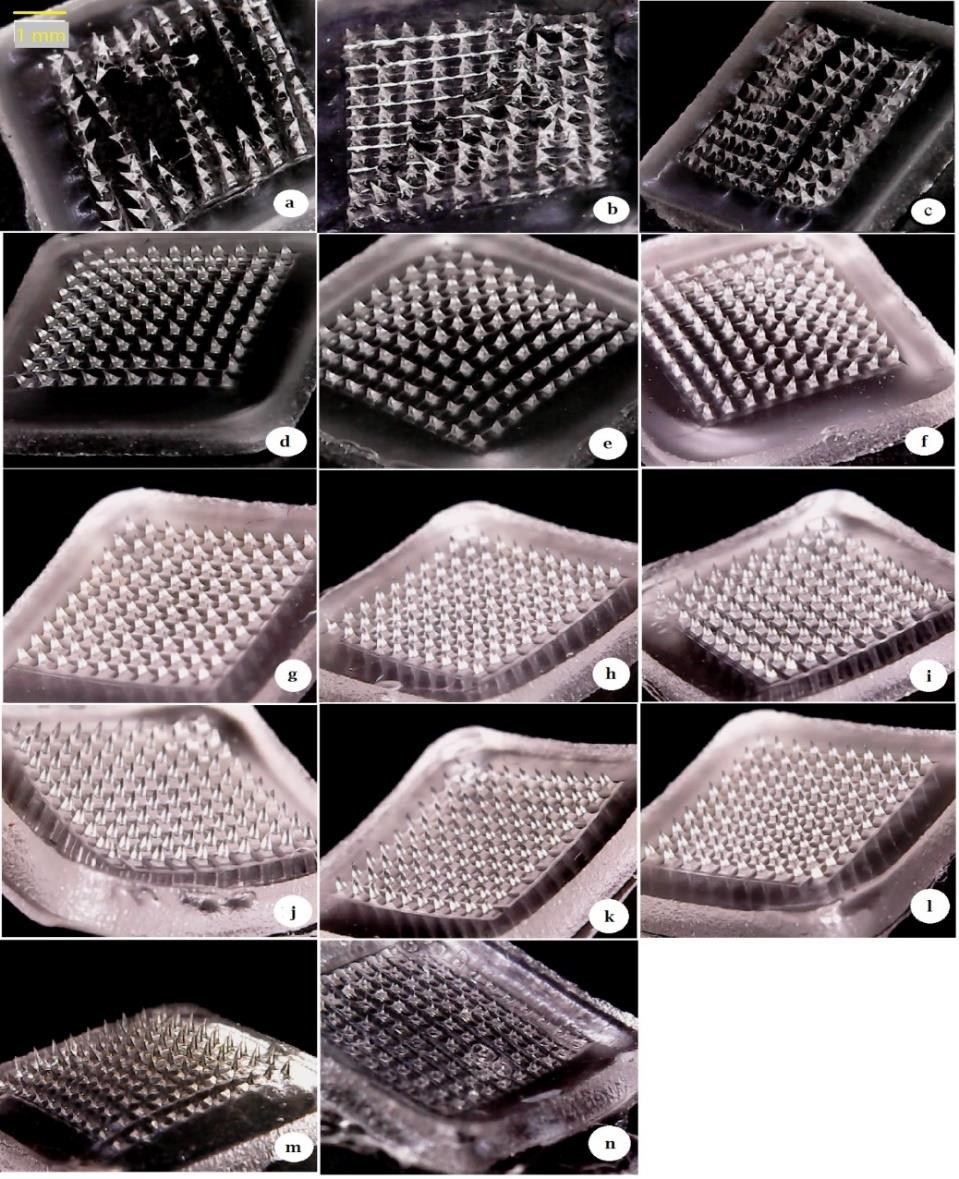
Digital microscope images of MN arrays prepared using different ratios of HA, where (a) and (b) represent F1, (c) F2, (d) F3, (e) F4, (f) F5, (g) F6, (h) F7, (i) F8, (j) F9, (k) F10, (l) F11, (m) F12 and (n) F13.

Formulations of F1 and F2 with a smaller concentration 5 % w/v of HA containing 1 % v/v and 2 % v/v of Tween 80, respectively, resulted in soft, elastic formulations with a gummy texture that are unsuitable for the MNs formation. Formulas from F3 to F11 have resulted in the formation of elegant shaped MN arrays that are fully formed. A digital microscope was used for the visualization of the fabricated MNs as illustrated in Figure 1. F12 and F13 formulas containing 55 % and 60 % w/v of HA, respectively, did not result in fully formed MNs. This can be attributed to the polymeric solution used, which was too viscous to fill the micron-sized cavities in the mould and therefore a limited number of needles were eventually formed.

### Characterisation of HA microneedle arrays containing LT-4 sodium

#### Measurement of the weight and thickness of the microneedle arrays

The MN arrays demonstrated a weight range of (4-55 mg) depending largely on the amount of HA added. The thickness values were in the range of (1.01 – 1.04 mm). Thus, the MN arrays possess a lightweight and delicate thickness suitable for convenient use by the patients. Detailed results regarding the weight and thickness of each formula are shown in the supplementary materials.

#### Assessment of the mechanical properties of microneedle arrays

To allow consistent application and drug delivery, MNs must be inserted into the skin without failure [33]. The mechanical strength of the prepared dissolving MNs was determined by evaluating their resistance to compression when an axial force was applied. This is a commonly used method to evaluate the mechanical properties of MNs. The applied axial force was 32N, which refers to the maximum force applied by a human during the manual insertion of MNs [18].

The results revealed a reduction in post-compression height, bending in certain needles, and needle fracture in some formulas. F1 and F2 resulted in the formation of delicate MNs, that were easily broken when moved before subjection to compression. F3-F7 showed needle fracture upon exposure to compression. F8, F9, F13 and F11 revealed MNs bending rather than fracturing. This refers to their ability to withstand the applied force better than the rest of the formulas. A higher concentration of HA polymer has resulted in a relatively stronger MN array. F11 was the most successful formulation yielding completely formed and strong MN arrays with the lowest height reduction percentage. The digital microscope images of MN arrays after exposure to compression using a Texture Analyser instrument were provided in the supplementary materials.

The degree of height reduction of MNs for different formulations was varied considerably since it is largely reliant on its unique components. The percentage of height reduction after compression for each MN array formula is shown in Table 3. The percentage of height reduction was observed to be inversely proportional to the concentration of the HA used. The mechanical strength increased by adding more polymer and an enhancement of elasticity was achieved by further addition of Tween 80. In addition to the role of Tween 80 in enhancing the solubility of LT-4 sodium, plasticizers like Tween 80 are also commonly used to minimize the intramolecular bonding between polymer chains. This provides the desired mechanical film qualities by boosting flexibility and preventing cracking, which is frequently employed in the transdermal drug delivery field [34].

The viscosity of the polymeric solution is proportional to the amount of polymer utilized. The higher the concentration of polymer, the more viscous the polymeric solution is leading to the formation of stronger MNs. It is critical to choose the right polymer concentration for microneedle shaft manufacturing; the thickness of the baseplate that supports the microneedles has been diminished by decreasing the polymer content. As a result, the needles were subjected to an extra-axial applied force which explains why mechanical strength decreased by reducing polymer concentration [35]. The mechanical strength evaluation and insertion study are virtually linked. Since they are related, the crucial aspect of efficient insertion is based on the ability of the MNs to endure the applied pressure [36].

The standard MN array height was found to be 432 ± 6.4 μm and it was used for the comparison of height reduction with other arrays subjected to compression.

#### *In vitro* drug release study

**Figure 2.**
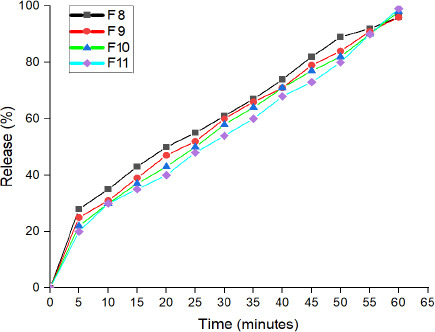
LT-4 sodium release profile from F8, F9, F10 and F11 MN arrays.

*In vitro* cumulative drug release percentages for the chosen formulas (F8, F9, F10 and F11) over 1 hour are illustrated in Figure 2. The graph demonstrates that the release profile from the different formulations is almost similar, with a non-significant difference between them (*P* > 0.05).

The release of LT-4 sodium was initiated within the first 5 minutes as the MNs’ tips became hydrated and began to dissolve by contact with water. After that, the dissolution of MNs increased with a simultaneous increase in LT-4 sodium release over an hour. During this hour, the MN arrays were diminished gradually until they eventually vanished after one hour.

Although there is a non-significant difference in the release profiles between different formulations, it can be observed that the release of LT-4 sodium from F8 and F9 was relatively higher than F10 and F11. This may be due to the presence of a higher ratio of HA in F10 and F11. After 1 hour, the release of LT-4 sodium from different MN arrays has reached 96-98 %.

The amount of the drug released from the four MN arrays has reached 20-25 % five minutes following the start of the test. This might be attributed to the drug adsorption on the surface of the polymeric MNs, besides to the hydrophilic property of HA polymer that will quickly dissolve in water and release drugs [37]. Following the complete dissolution of the needles’ tip within 5 minutes, a slower rhythm of drug release has been noticed. This is because the backing layer may act as a reservoir for drug release.

#### Detection of microneedles insertion ability using Parafilm M^*®*^

**Figure 3.**
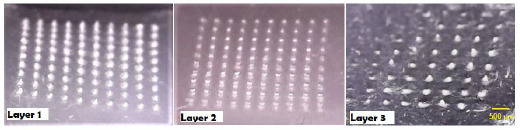
Insertion test using Parafilm M^®^ layers by F11 MN array that clearly shows the successful penetration through the 3^rd^ layer.

Eight layers of Parafilm M® were employed to mimic the skin layers in order to assess the depth of penetration of MNs in the skin. As reported by Larrañeta *et al*., penetration is regarded as efficient if greater than 20 % of micro conduits are created in each layer [18]. Thus, the insertion test findings indicate that MNs are located between the third and fourth layers, as shown in Figure 3.

**Figure 4.**
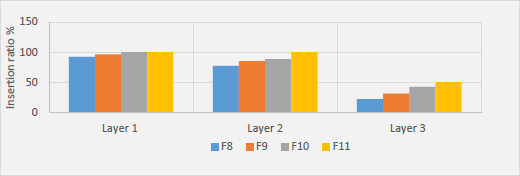
Illustration of the insertion ratio of the MN arrays (F8, F9, F10 and F11) created in Parafilm M^®^ sheets.

Moreover, the thickness of one Parafilm M® layer is approximately 126 ± 7 μm, implying that the MNs were penetrated up to 378 μm depth (approximately 87 % of needles height). As demonstrated in Figures 3 and 4, F11 MN arrays showed 100 % penetration in the first and second layers and 50 % in the third layer. This indicates their adequate mechanical strength with the sharpness of the tips required for efficient penetration across the skin tissue barrier. F8, F9 and F10 have exhibited a lower number of micro holes, especially in the third layer as illustrated in Figure 4, proposing lower mechanical properties probably due to the lower concentration of HA polymer contained in their MN arrays.

#### Insertion of microneedle arrays into excised human skin

**Figure 5.**
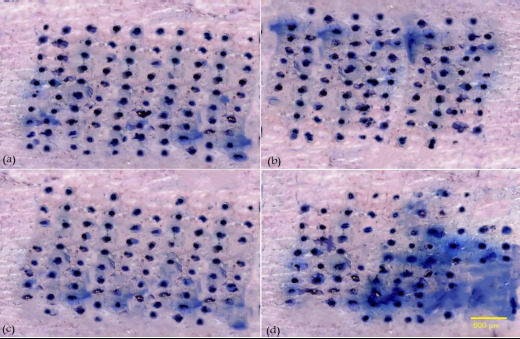
Digital microscope images of human skin following the application of HA MN arrays and staining with methylene blue dye showing the pores formed by different formulas, where (a) represents F11, (b) F10, (c) F9 and (d) F8.

To assess the MNs’ effectiveness in the penetration of the uppermost layer of the skin (*stratum corneum*), an insertion study using human skin was performed. Figure 5 shows the human skin stained with methylene blue dye after treatment with HA MN arrays. The pores created by the MNs were stained with the dye, whereas the intact skin remained unstained.

The 10 × 10 MN arrays with varying concentrations of HA (F8, F9, F10 and F11) resulted in a 75 to 100 % insertion ratio after 1 minute of application of MN arrays. These findings highlight the capability of the MN arrays to penetrate the skin effectively [32]. The insertion ratio for each sample was estimated by dividing the number of blue spots on the skin after insertion by the number of needles in the arrays [16].

The insertion ratio of MN arrays was increased with an increase in their HA content. This can be related to the higher mechanical strength achieved by increasing the concentration of HA in each formula. Consequently, MN formula F11 demonstrated a 100 % insertion ratio due to their higher HA concentration (50 % w/v), resulting in the greatest mechanical strength required for efficient skin penetration.

MN arrays were inserted into the skin using gentle thumb pressure. Different types of applicators have been designed to minimize variation in insertion forces for the same MN array [38,39]. When appropriately engineered, the consistency of the applied force can be better with an applicator than with a thumb. Yet, inserting MNs into skin using a thumb force has been a typical technique of administration and multiple groups have clearly confirmed uniform and full MN array insertion while using this approach [40-42].

### Characterisation of the optimum formula

HA MN array formula F11 was identified as the optimum formula because it showed the required mechanical strength, high drug content (data not shown) and superior penetration into the skin surface. (i.e. highest insertion capability), in addition to its ease of formulation during the fabrication process and elegant appearance. Therefore, the F11 formula was subjected to further investigations to ensure its eligibility as a transdermal drug delivery system for LT-4 sodium.

#### Histological examination of MN treated skin

**Figure 6.**
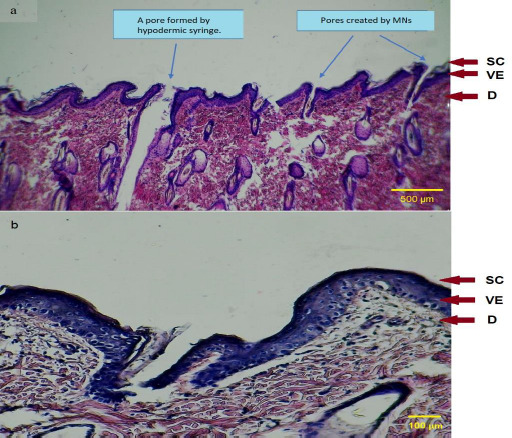
Digital microscope images of cross-sectioned human skin samples stained with H&E for MN treated skin (a) at power 4x and (b) at power 10x; demonstrating the location of microchannels within the skin in comparison to the hypodermic needle. Where: (SC) represents *stratum corneum*, (VE) viable epidermis and (D) dermis.

To demonstrate the penetration depth of the microchannels within skin layers achieved by the application of MN arrays, histological examination of the cross-sectioned and stained human skin samples was performed as shown in Figures 6 (a) and (b). It can be observed that F11 MN arrays following the insertion into the skin have developed drug microchannels (drug permeation routes) through the different skin layers, including the *stratum corneum*, viable epidermis and dermis.

These microchannels formed in the skin showed approximately similar shapes to the inserted MNs arrays indicating their uniform insertion. It is believed that these microchannels play a significant role in the permeation of LT-4 sodium into the systemic circulation by bypassing the *stratum corneum* barrier. In addition, Figure 6 (a) highlights the difference in size between pores created by the MN arrays and by the traditional hypodermic syringe.

The pores formed by hypodermic needles are obviously larger, deeper reaching the deep dermis and having a greater opportunity to stimulate the nerve endings than those formed by MNs. Ultimately, these observations suggested that F11 MN arrays have the potential for successful transdermal delivery of LT-4 sodium across the skin with a little tendency to trigger the nerve endings located at the deep dermis layer [43].

#### *In vivo* dissolution of MN arrays

**Figure 7.**
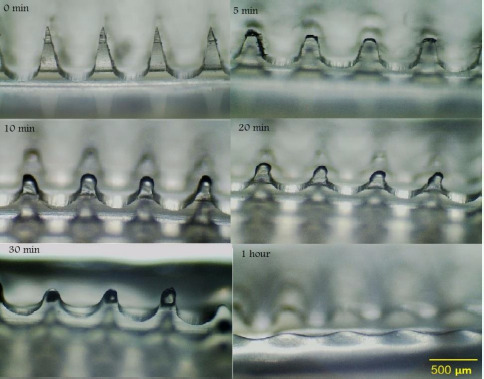
Time-course images of F11 MN arrays after being applied to the skin of a live rat and withdrawn at the indicated time points.

The images of the *in vivo* dissolution study of F11 MN arrays within rat skin are illustrated in Figure 7. As depicted in the images, the dissolution of the MN arrays was very fast, starting 5 minutes after insertion into the skin, with complete dissolution achieved by 1 hour. The fast dissolution of F11 MNs suggests rapid delivery of LT-4 sodium across the skin layers.

It has been observed that HA MNs possess self-dissolving properties and are easily dissolved upon application to the skin [20]. This rapid dissolution is caused by the high hydrophilicity of HA [44]. In their research study, Zhuangzhi Zhu *et al*., have shown that HA is a suitable candidate for dissolving MNs, as it starts dissolving rapidly upon application into the skin, proposing that it required 60-120 minutes for their needles to be completely dissolved in the skin [44].

#### Visualization of microneedles by scanning electron microscopy (SEM)

**Figure 8.**
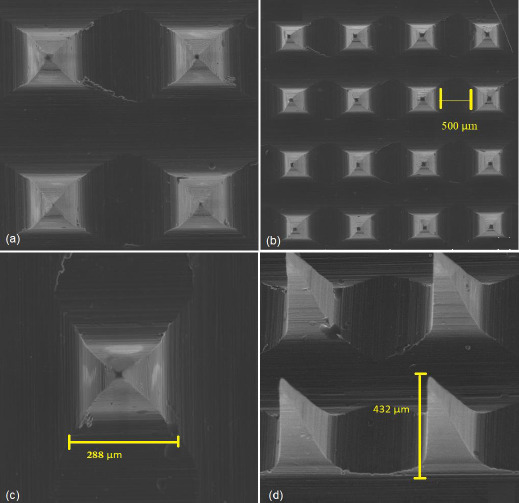
SEM images of HA MN array F11, where: (a) top view of MN array at a magnification of 200x, (b) A single array of pyramidal-shaped MNs shows the distance between two needles at a magnification of 100x, (c) top view of a single needle in the array showing the base width at a magnification of 300x, and (d) lateral view of MNs showing the needle length at a magnification of 200x.

The SEM images of F11 MN array are demonstrated in Figure 8. The images highlight the proper formation of sharp, pyramidal-shaped and equally interspaced MNs. Additionally, the images elucidate the dimensions of MNs. For example, an individual MN from the array showed a mean length of 432.0 ± 6.4 μm. The needle tip diameter of 9.8 ± 1.3 μm, base diameter of 288 ± 2.2 μm and interspacing distance (the distance between two needle tips) of 500.0 ± 5.5 μm. Based on these dimensions, the MNs possess sharp tips with a strong base required for successful penetration into the skin [32].

According to the SEM images, the prepared HA MN by micromoulding technique were shorter than the master structure (PDMS mould), which is 500 μm needle length. This can occur due to the difference in polymer filling capability into the mould, which is based on the polymer viscosity and the shrinkage of polymeric solution during drying [45]. It is anticipated that this reduction in the length of MNs does not greatly influence the transdermal delivery of LT-4 sodium since they showed the excellent formation of microchannels through the different skin layers, including the dermis where the drug can be systemically absorbed.

#### *Ex vivo* permeation study

The transdermal delivery of LT-4 sodium is limited due to its low permeability characteristics through the *stratum corneum* layer. MNs may enable LT-4 sodium delivery through the skin by physically disrupting the *stratum corneum* barrier and transferring molecules through the micro-channels into the dermis microcirculation. Therefore, to investigate the influence of MNs in enhancing transdermal delivery of LT-4 sodium through the skin, an *ex vivo* permeation study using human skin was carried out.

**Figure 9.**
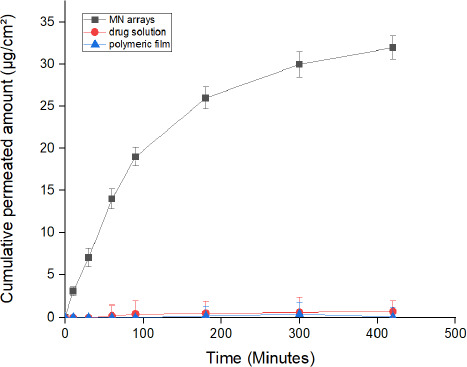
The cumulative amount of LT-4 sodium permeated across human skin in μg/cm^2^ from HA MN arrays F11 in comparison to control preparations (aqueous drug solution and polymeric film). Data is presented as the mean ± SD (n = 6).

HA MN array formula F11 was used in this permeation study since it is regarded as the optimum formula, as shown previously. In addition, to the use of F11 MN arrays in the permeation study, control formulas of LT-4 sodium as an aqueous solution and as a polymeric film without needles (needle-free) were utilized for comparison purposes. The cumulative amount of LT-4 sodium permeated from MN array F11, drug solution and the polymeric film is demonstrated in Figure 9. As can be observed from the graph, the microneedle array F11 has significantly (P *≤* 0.05) increased LT-4 sodium permeation through the skin (cumulative permeated amount of 32 ± 2 μg/cm^2^) in comparison to solution control (cumulative permeated amount of 0.7 ± 0.07 μg/cm^2^) and the polymeric film (0.1 ± 0.02 μg/cm^2^) over the period of 7 hours.

The MNs arrays have delivered 64 % ± 4 of their loading dose over 7 hours period, whereas the control preparations of aqueous solution and polymeric film were only able to deliver 1.4 % ± 0.14 and 0.2 % ± 0.04 of total loading dose, respectively, during the same period. This represents an approximately 46-320 fold increase in delivery of LT-4 sodium than that was achieved with the control preparations (aqueous solution and polymeric film, respectively).

Moreover, as the drug was encapsulated within MN arrays, the lag time was less since the drug began to diffuse quickly upon application of MNs. In this study, the HA MNs were started to dissolve within 5 minutes of application into the skin, supporting the shortening in lag time. The lag time for the MN arrays was only 10 minutes, while the drug solution (control) lag time was 44 minutes and the polymeric film lag time was 120 minutes. This enhancement of LT-4 sodium delivery is related to the ability of MNs to bypass the *stratum corneum* barrier by creating microchannels through the skin to facilitate drug permeation. In addition, the hydration of the drug reservoir in the polymer matrix is enhanced by the fluid withdrawn from the skin via the microchannels leading to an improvement in drug diffusion [40]. The results obtained from this permeation study suggest the successful application of HA MN arrays in the delivery of LT-4 sodium. This can be considered a promising approach when used in humans since it may improve LT-4 sodium bioavailability.

#### Skin irritation test

**Figure 10.**
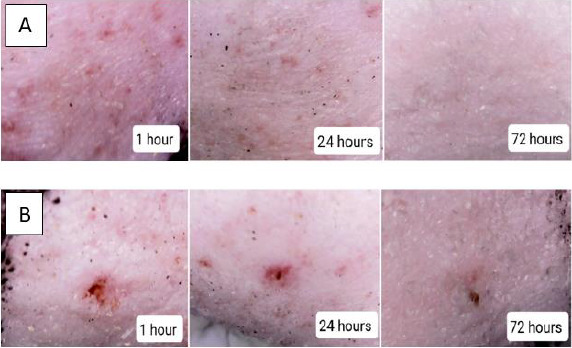
Images of skin irritation following the application of (a) HA MN arrays F11 and (b) conventional hypodermic syringe on rat skin at 1 h, 24 h and 72 h.

 In the skin irritation test, the scores for erythema and edema from the skin sites treated with MN arrays were evaluated for the rats at 1, 24 and 72 hours [46]. As shown in Figure 10, slight erythema but no edema appeared at the application sites at one hour and disappeared within 24 hours in comparison with a hypodermic needle.

The P.I.I. of the MN arrays was calculated to be 1.7. The slight redness that was seen on the skin site one hour following the removal of the MNs, suggests that the physical compression during the application of MNs may cause this skin redness. However, this redness disappeared 24 hours and 72 hours after the application of MNs. Therefore, these findings and according to Draize dermal scoring criteria indicated that the irritation and skin damage caused by HA MN arrays were slight [27]. Whereas the hypodermic syringe has resulted in bleeding and a dramatic scar which was obvious even 72 hours following the application of the hypodermic needle.

## Conclusions

The current study revealed for the first time the potential of HA dissolving MN arrays for efficient transdermal delivery of LT-4 sodium by overcoming the *stratum corneum* barrier. Additionally, this work clearly demonstrates the benefits of HA MN arrays in enhancing skin permeation of drugs with poor permeability characteristics. The LT-4 sodium loaded HA MN arrays represent a minimally invasive system that showed excellent skin insertion with rapid dissolution and release of LT-4 sodium and superior transdermal delivery in comparison to control preparations. Thus, they may provide a feasible and convenient alternative approach for oral administration, which is accompanied by variable rate and extent of LT-4 sodium absorption. However, further *in vivo* studies are required to confirm the MN arrays’ efficacy and safety for clinical use.



## Figures and Tables

**Table 1.** Composition of polymeric dissolving microneedle formulations.

Formulation Code	LT-4 (% w/v)	HA (% w/v)	Tween 80 (% v/v)	DW (% v/v) Up to
F1	0.05	5	1	100
F2	0.05	5	2	100
F3	0.05	10	1	100
F4	0.05	15	1	100
F5	0.05	20	1	100
F6	0.05	25	1	100
F7	0.05	30	1	100
F8	0.05	35	1	100
F9	0.05	40	1	100
F10	0.05	45	1	100
F11	0.05	50	1	100
F12	0.05	55	1	100
F13	0.05	60	1	100

**Table 2.** Draize dermal scoring criteria [27]:

P.I.I.	Classification
0.0-0.4	No irritation
0.5-1.9	Slight irritation
2.0-4.9	Moderate irritation
5.0-8.0	Sever irritation

**Table 3.** Mean height, height percentage and height reduction percentage after compression for each MN array formula. Data is presented as the mean ± SD

Formula code	Mean height (μm) after compression	Height % after compression	Height reduction %
F3	277.65 ± 4	67.80 ± 4	32.24 ± 2
F4	292.93 ± 2	68.70 ± 8	31.30 ± 4
F5	326.17 ± 5	75.50 ± 4	24.51 ± 6
F6	349.56 ± 6	80.91 ± 5	19.09 ± 8
F7	367.44 ± 4	85.05 ± 4	14.95 ± 3
F8	382.44 ± 8	88.52 ± 2	11.48 ± 2
F9	399.72 ± 7	92.52 ± 3	7.48 ± 5
F10	402.76 ± 8	93.23 ± 5	6.77 ± 6
F11	411.22 ± 4	95.18 ± 2	4.82 ± 4
