# Supplementary material for: Levothyroxine sodium loaded dissolving microneedle arrays for transdermal delivery
Source: ADMET DMPK. 2022 Sep 13;10(3):213–30. doi: 10.5599/admet.1317 (PMC9484701; doi:10.5599/admet.1317)
Supplement: Supplementary file 1 [file Admet-10-1317_S1.pdf]

Supplementary Material

Levothyroxine sodium loaded dissolving microneedle arrays for transdermal delivery

Riyam F. Ghazi, Mohammed H. Al-Mayahy*

Department of Pharmaceutics, College of Pharmacy, Mustansiriyah University, Baghdad-Iraq

ADMET and DMPK (2022) doi: https://doi.org/10.5599/admet.1317

Table S1: Mean weight, thickness and pH of the prepared MN arrays.

Formula code	Weight (mg)	Thickness (mm)	pH
**F1**	4.10	1.021	7.24
**F2**	5.72	1.023	7.20
**F3**	12.31	1.014	7.20
**F4**	17.65	1.026	7.17
**F5**	21.28	1.037	7.26
**F6**	25.72	1.024	7.16
**F7**	33.81	1.042	7.22
**F8**	38.89	1.041	7.16
**F9**	43.75	1.033	7.11
**F10**	51.21	1.036	7.29
**F11**	52.39	1.045	7.28
**F12**	52.97	1.043	7.32
**F13**	55.41	1.046	7.12

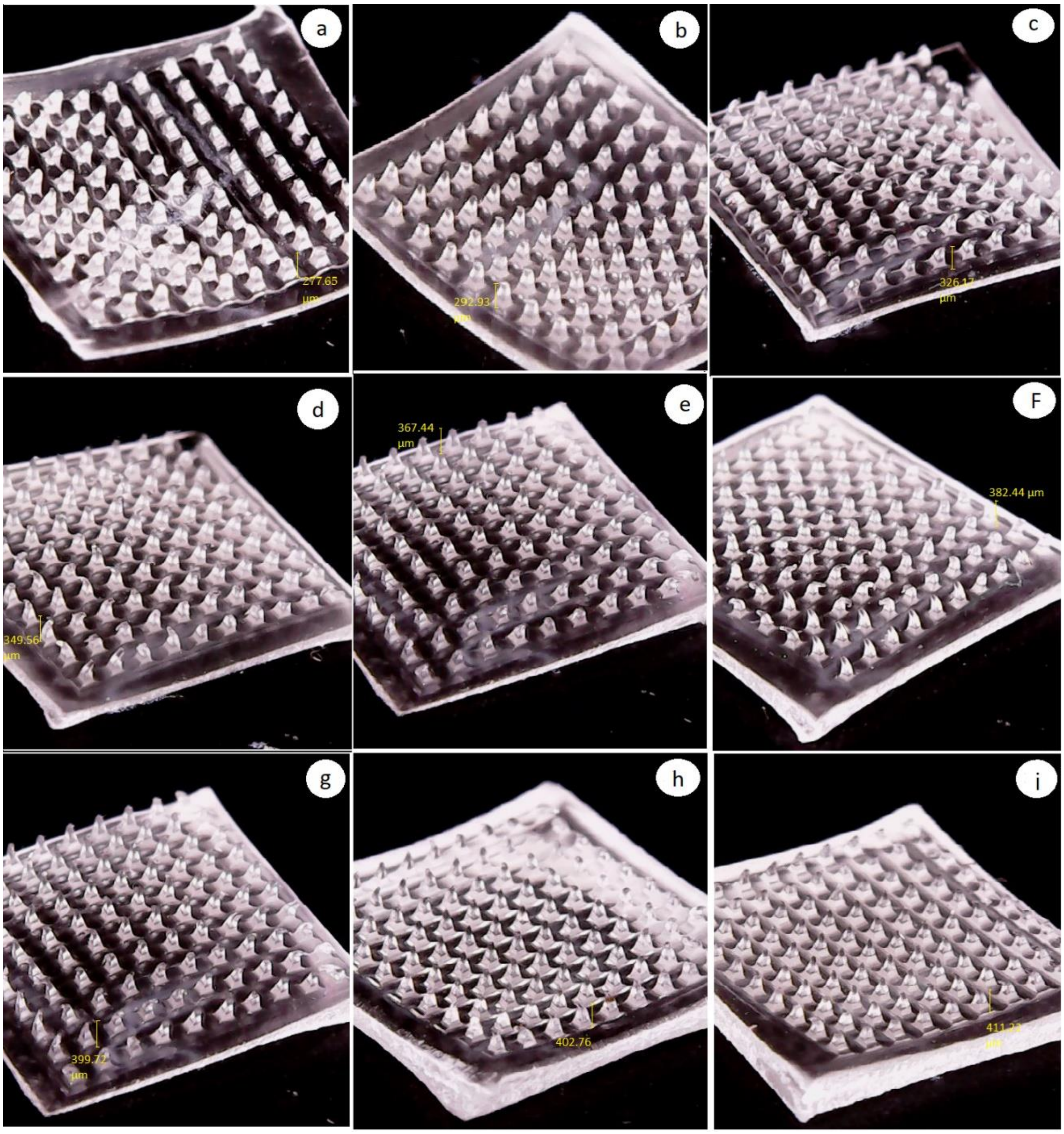

Figure 1S. Digital microscope images of MN arrays after compression using texture analyser, where (a) represents F3, (b) F4, (c) F5, (d) F6, (e) F7, (f) F8, (g) F9, (h) F10 and (i) F11.
